# Potential of Immune-Related Therapy in COVID-19

**DOI:** 10.3389/fphar.2020.609212

**Published:** 2021-01-26

**Authors:** Chunjue Yuan, Ruoyun Li, Guohong Liu, Yunbao Pan

**Affiliations:** ^1^ Department of Laboratory Medicine, Zhongnan Hospital of Wuhan University, Wuhan University, Wuhan, China; ^2^ Department of Radiology, Zhongnan Hospital of Wuhan University, Wuhan University, Wuhan, China

**Keywords:** SARS-CoV-2, COVID-19, vaccine, cytokine storm, convalescent plasma, mesenchymal stem cell

## Abstract

At the beginning of 2020, a sudden outbreak of new coronavirus, severe acute respiratory syndrome coronavirus 2 (SARS-CoV-2), infections led to anxiety, panic, and crisis among people worldwide. The outbreak first occurred in Wuhan, China, in late December 2019 and then spread rapidly across the globe, thus becoming a major public health emergency. Although the current epidemic situation in China tends to be stable, coronavirus disease 2019 (COVID-19) continues to spread globally. At present, no specific therapeutic drugs and vaccines are available against COVID-19. Also, the pathogenesis of the SARS-CoV-2 is not fully clear. Human immunity is important in SARS-CoV-2 infection. Studies have shown that excessive inflammation caused by SARS-CoV-2 infection and subsequent induced uncontrolled cytokine storm are the main causes of disease deterioration and death of severe patients. Therefore, immune-related research is of great significance for the prevention, control, and prognosis of COVID-19. This study aimed to review the latest research on immune-related treatment of COVID-19.

## Introduction

In late December 2019, several patients with pneumonia of unknown etiology were detected in Wuhan, China. They were diagnosed with viral pneumonia, and the pathogen was subsequently isolated from patient samples. Electron microscopy showed that the pathogen presented a typical coronavirus morphology and was different from the coronavirus found in the past, which attracted great attention from the Chinese Center for Disease Control and Prevention, and an emergency response was urgently initiated. The viral genome sequence was first published on January 11, 2020, and confirmed as a completely new coronavirus named 2019 new coronavirus (nCoV) through sequence analysis and etiological studies (Zhu N et al., 2020). On February 11, 2020, the International Committee on Taxonomy of Viruses officially named the new coronavirus (2019-nCoV) severe acute respiratory syndrome coronavirus 2 (SARS-CoV-2) considering the phylogeny and taxonomy of the virus (Gorbalenya et al., 2020). On the same day, the World Health Organization announced that the disease caused by this virus was called coronavirus disease 2019 (COVID-19) (WHO, 2020a). Although most patients are mild to moderate and have a good prognosis, the mortality rate of critically ill patients is high. Therefore, cellular immunity and neutralizing antibodies are important in protection against infection with SARS-CoV-2. Anti-SARS-CoV-2 vaccines must induce strong cellular immunity and a high titer of neutralizing antibodies to fully protect vaccinated individuals. As for the lethal process of neocoronavirus infection, a hyperinflammatory syndrome known as secondary hemophagocytic lymphohistiocytosis (sHLH) appeared among part of COVID-19 patients because of cytokine storm, eventually leading to multiorgan failure (Mehta et al., 2020; Ruan et al., 2020); thus, finding solutions to prevent and reverse cytokine storm is also the key to the rescue of severe COVID-19 patients (Huang et al., 2020). Since the outbreak of the epidemic, scientific researchers from all walks of life have devoted themselves to exploring a potential treatment for COVID-19. A management algorithm, which correlated the clinical features with laboratory and imaging findings, has been established for more appropriate diagnostic and therapeutic strategies (Galluccio et al., 2020). And current potential antiviral therapies are divided mainly into two aspects based on their action targets: 1) directly targeting the SARS-CoV-2 and 2) modulating immune cells or system. This study summarized mainly the research progress of potential treatment related to immunity.

## Treatment Against COVID-19

Up to August 10, 2020, more than 19.71 million people were diagnosed with COVID-19, leading to 720,000 deaths. As a safe and effective antiviral treatment against this epidemic is urgently needed, a series of studies are ongoing, including prevention and therapies.

### Preventive Vaccines

Historic experience has proven that vaccines are the best way to prevent infectious diseases, but qualified vaccines need to undergo extensive trials to ensure their safety and effectiveness. A preclinical animal model test is required after the vaccine is developed, and a clinical trial can be declared only when the safety and effectiveness are ensured. Among these, the primary aim of phase I clinical evaluation is to explore the safety of the vaccine; the main purpose of phase II clinical evaluation is to report the efficacy and general safety information of the vaccine in the target population; and the purpose of phase III clinical evaluation is to comprehensively evaluate the protective effect and safety of the vaccine, which requires 1–4 years. Hundreds to thousands of volunteers were followed up for these assessments. Vaccine development against COVID-19 mainly focuses on viral vector, nucleic acid, and inactivated and recombinant proteins at present.

#### Viral Vector Vaccine

##### Adenovirus Recombinant New Corona Vaccine

After developing the Ebola vaccine with adenoviral vectors previously, the Academy of Military Medical Sciences and Tianjin CanSino Biological Inc. have rapidly developed a recombinant new coronavirus vaccine (Ad5-nCoV) with adenoviral vectors. Its safety and effectiveness have been verified in animal-related tests. On March 16, 2020, it became the first vaccine against the SARS-CoV-2 to enter the clinical trial in China (ChiCTR2000030906/NCT04313127). The vaccine carries spike (S) protein using modified human adenovirus type 5 (Ad5), leading to the neocoronavirus invasion (Chen Y et al., 2020). The immune system recognizes the virus antigen and develops an immune memory of S protein after the Ad5 vaccine injection. When attacked by the SARS-CoV-2, the memory immune system immediately recognizes and produces corresponding antibodies, thus achieving viral eradication. The 108 volunteers vaccinated in the phase I trial were mainly permanent residents of Wuhan and aged between 18 and 60 years. These volunteers were regularly followed up to determine whether adverse reactions occurred and whether anti-S protein-specific antibodies were produced *in vivo*. So far, 108 vaccinated volunteers have been in good health, and the overall progress of the phase I trial has been smooth. The phase II clinical trial of the Ad5-nCoV, which aimed at evaluating the immunogenicity and safety of the vaccine, was carried out in Wuhan among 508 volunteers with a broader age range on April 12 (ChiCTR2000031781/NCT04341389). Further, 250 participants aged more than 18 years were supposed to be in the middle-dose vaccine group, and 125 were in the low-dose and placebo groups, respectively. Immunogenicity was tested on days 0, 14, and 28 and 6 months after vaccination without hospitalization. The results of another clinical trial consisting of 696 participants (NCT04398147), conducted by CanSino with the Canadian partner, will also be revealed in December 2021. The global phase III clinical trial of the Ad5-nCoV, manufactured by CanSino and Beijing Institute of Biotechnology, started recruiting 40,000 volunteers of healthy adults aged 18 years old and above (NCT04526990). However, some risks are involved. If the human body has been previously infected with adenovirus type 5, it will produce its relevant antibodies, which will attack the vector rather than express the S protein if vaccinated with the recombinant vaccine, thereby rendering the vaccine ineffective (Zhu F-C et al., 2020; Shi et al., 2020).

### AZD1222

The adenovirus vector vaccine AZD1222 (formerly known as ChAdOx1 nCoV-19) developed by AstraZeneca United Kingdom and Oxford University under the “Warp Speed” program is currently undergoing phase I/II clinical trials (NCT04324606) in the United Kingdom and phase III clinical trials in Brazil and South Africa. In phase I/II clinical trials (*n* = 1077, with an average age of 35 years) between April 23 and May 21, 543 participants were injected with AZD1222 and the other 534 received the meningococcal conjugate vaccine. Transformed from attenuated adenovirus, this replicator-defective chimpanzee virus vector carries a transgene that encodes the S protein of novel coronavirus. Patients will produce the S proteins after vaccination, thus triggering the immune system to produce antibodies against the virus. The results of phase I/II support the ongoing phase III clinical trial on a larger scale, as they have certified safety and immunogenicity of AZD1222, indicating its potential role in obstructing the pandemic of COVID-19 (Folegatti et al., 2020).

#### Nucleic Acid Vaccine

##### mRNA Vaccine

###### BNT162

BNT162, BioNTech’s novel coronavirus vaccine candidate, went into clinical trials in late April and then entered overseas clinical trials in cooperation with Pfizer and Fosun Pharma. BNT162 includes four vaccine candidates: BNT162a1, BNT162b1, BNT162b2, and BNT162c2. On July 1, Pfizer posted on medRxiv about the positive results of phase I and part of phase II clinical trials of BNT162b1, which is the first published clinical trial based on an mRNA vaccine technology. BNT162b2, one of the promising candidates for COVID-19, is a full-length S protein mRNA granted fast-track status by the Food and Drug Administration recently. It has been proved in phase I/II trials to induce T cells for better recognition of S epitopes, thus contributing to immune responses in older adults (NCT04380701) (Mulligan et al., 2020). The phase III clinical trial, in approximately 150 sites in six countries including 39 US states, has proved the safety and efficacy of BNT162b2 up to 95%. Revealed data demonstrated its good tolerance across all populations with 43,611 enrolled participants, and less older vaccinated people were reported with milder adverse events, which is consistent with an earlier study (Polack et al., 2020). Companies are planning to submit to the FDA for emergency use authorization (EUA) and share data with other regulatory agencies worldwide.

### mRNA-1273

The phase I clinical trial of mRNA vaccine mRNA-1273, developed by the National Institutes of Health and Moderna, was officially launched on March 16 (NCT04283461). A total of 120 healthy volunteers, aged between 18 and 55 years, were recruited within 6 weeks to assess the safety and immunogenicity of mRNA-1273 (Laura and Johnson, 2020). This dominant vaccine also targets the S protein of the SARS-CoV-2. The S protein, which encodes the SARS-CoV-2, is delivered by lipid nanoparticles (LNPs) and regulated by mRNA, activating an immune response and thus curtailing the invasion of new coronavirus. In the phase I study, participants injected with mRNA-1273 twice developed robust neutralizing antibodies, whose neutralizing activity against SARS-COV-2 was reserved after 42 days. ModernaTX, Inc. started phase II trials on May 29, 2020, among 600 volunteers with doses of 50 mcg (NCT04405076), while phase III trials are supposed to recruit 30,000 healthy adults from 89 clinical trial spots throughout the United States. Volunteers will be randomly assigned to either a test group or a control group with a 100 µg dose of mRNA-1273 vaccine or a placebo saline injection, followed by a second dose 28 days later (NCT04470427). Compared with traditional vaccines, this vaccine is superior in a short development cycle, with no viral activity and good safety, and is expected to be used for COVID-19 prevention (Anderson et al., 2020).

## DNA Vaccine

### New Coronavirus Candidate DNA Vaccine INO-4800

On April 6, 2020, INO-4800, developed by INOVIO Pharmaceuticals, became the first new coronavirus candidate DNA vaccine to enter clinical trials. DNA vaccines insert specific antigen genes into eukaryotic expression vectors, directly injected or encapsulated by liposomes into the body, and their corresponding antigen proteins are expressed in the cells, inducing specific humoral and cellular immune responses, thus activating the body’s immune system to achieve COVID-19 prevention. In phase I clinical studies during April (NCT04336410), INO-4800 has shown a robust neutralizing antibody and T-cell immune response to the virus in 40 enrolled participants aged 18–50 years; its phase I/II clinical effectiveness studies will also be performed by the Korean group in the summer of 2020 (NCT04447781) (Trevor et al., 2020).

### GX-19

Genexine led the research on GX-19 on March 13, 2020, which was the first COVID-19 DNA vaccine with Korean FDA approval for phase I/IIa clinical trials (NCT04445389). It entered clinical trials on June 17; 60 healthy volunteers were injected in a two-dose trial during phase I. The trial was supposed to finish in 3 months. Later in September, phase IIa clinical trials among 150 participants (including placebos) were started to assess the safety and efficacy of GX-19, and the estimated completion date will be March 17, 2021 (Ye et al., 2020).

#### Inactivated Vaccine

##### PiCoVacc Vaccine

PiCoVacc vaccine, a polyvalent vaccine prepared by inactivating and purifying the virus using *β*-propiolactone, induces SARS-COV-2-specific neutralizing antibodies in mice, rats, and nonhuman primates as proposed by Qin et al., broadening a possible neutralization capability for the virus circulating globally (Gao et al., 2020). A majority of antigens and epitopes of the virus aim at conserved epitopes, thus reducing virus escape. Sinovac Biotech Co. has enrolled 144 volunteers aged 18–59 years for phase I and 600 for phase II (NCT04352608).

#### Recombinant Protein Vaccines

Pathogen-specific protein genes are inserted into appropriate expression systems via genetic engineering after recognizing their immunogenicity (such as *E. coli* yeast microbes). Proteins are cultivated on a large scale *in vitro* and further purified to become vaccines. Recombinant protein vaccines account for the majority of resistance to COVID-19 to date (nearly up to 42%), targeting the S protein of this virus.

### NVX-CoV2373

Based on the gene sequence of COVID-19 (GenBank accession number, MN908947; nucleotides 21563–25384), Novavax generated an S protein antigen of this novel virus via its recombinant nanoparticle technology. Coupled with a unique saponin-based Matrix-M adjuvant, NVX-CoV2373 stimulates antigen presentation in local lymph nodes, enhancing the immune response and high levels of neutralizing antibodies. In preclinical trials, NVX-CoV2373 blocks the combination between S proteins and virus-targeted receptors. Clinical trials in phase I of NVX-CoV2373 recruited 131 participants aged 18–59 years (NCT04368988) to confirm its safety and immunogenicity and people aged 60–84 years are in need of expanding participant range in phase II trials up to 40 sites across Australia and/or the United States. This phase I/II trial is supposed to be complete on November 18, 2021 (Keech et al., 2020).

### Therapeutic Options

#### Corticosteroids in COVID-19

Studies have shown that critically ill COVID-19 patients can develop a systemic inflammatory response, and uncontrolled cytokine storm caused by SARS-CoV-2 infection subsequently leads to lung injury, multisystem organ dysfunction, and eventually death. Corticosteroid, a potent anti-inflammatory, antifibrotic, and vasoconstrictive drug, has been previously applied in many other pulmonary infections to alleviate the condition of patients (Bozzette et al., 1990; Stockman et al., 2006; Rodrigo et al., 2013). Recently, a retrospective cohort study in China also revealed a potential mortality benefit of corticosteroids in COVID-19 (Chaomin et al., 2020), providing new clues on therapeutic approaches to novel coronavirus.

A number of studies on corticosteroids’ role in SARS-CoV-2 infection have been carried out (Halpin et al., 2020; Peters et al., 2020). In the United Kingdom, a large open-label randomized trial recruited 6425 hospitalized patients with COVID-19. 2104 patients were treated with a 10-day course of dexamethasone (6 mg/d [oral or intravenous]) while the others received the standard of care, and mortality at 28 days showed the superiority in dexamethasone group (Horby et al., 2020). Meanwhile, Peter Horby et al. offered alternative options including prednisone 40 mg, methylprednisolone 32 mg, and hydrocortisone 160 mg (the total daily dose equivalencies to dexamethasone 6 mg). The World Health Organization (WHO) also performed a meta-analysis based on data from seven randomized clinical trials of corticosteroids in severe COVID-19 patients (PROSPERO CRD42020197242) (Sterne et al., 2020) and found that corticosteroids could reduce the risk of death in COVID-19 cases by 20%. WHO then issued living guidance for the use of corticosteroids in severe COVID-19 cases on September 2 (WHO, 2020b).

Though promising results were reported, putting corticosteroids into clinical use widely has been controversial (Bozzette et al., 1990; Stockman et al., 2006; Rodrigo et al., 2013, Prescott and Rice, 2020; Sanders et al., 2020). It is critical to weigh the potential benefits of corticosteroids against much potential harm associated with these drugs and clinicians should pay close attention to COVID-19 patients who are receiving corticosteroids for adverse effects and risk of reactivation of latent infections.

#### Convalescent Plasma Therapy

On the evening of February 13, Zhang Dingyu, president of Wuhan Jinyintan Hospital, publicly appealed for plasma donation from patients who recovered from new coronary pneumonia, as neutralizing antibodies in the plasma of recovered patients might help suffering patients (Wang Y et al., 2020). Thus, a clinical trial of convalescent plasma transfusion therapy was conducted on five severe patients by the team of Shenzhen Third People’s Hospital (Shen et al., 2020); the patients included two women and three men aged 36–65 years. Applied plasma came from another five convalescent patients during their 10–22 days of hospitalization. The progression of COVID-19 enhanced rapidly after antiviral treatment because the viral load level was continuously high, thus leading to acute respiratory distress syndrome (ARDS). Specific antibody immunoglobulin G (IgG) in convalescent plasma bound to the SARS-CoV-2 with an antibody titer higher than 1:1000 and a neutralizing antibody titer greater than 40. The body temperature returned to normal within 3 days and the arterial partial pressure of oxygen/oxygen uptake concentration (PaO2/FiO2) increased within 12 days, while the sequential organ failure assessment score decreased. The viral load also decreased to negative within 12 days with an increasing level of new coronavirus-specific antibody. The results suggested that the antibodies in the convalescent plasma might clear the virus and improve patient symptoms. The impact of the timing of plasma transfusion is not clear enough due to the small sample size and lack of a control group. Therefore, these observations remain to be evaluated in clinical trials.

Plasma therapy, also known as sclerotherapy, is the treatment of patients with corresponding diseases using the plasma, serum, or immunoglobulin from cured patients, which still has a large number of antibodies shortly after recovery. As a result, patients with the same disease can obtain passive immunity instantly through the input of exogenous antibodies. The safety, effectiveness, and applicability of this therapy have been controversial for a while. However, sclerotherapy is still new hope for the clinical treatment of the new coronavirus infection, considering previous research on SARS and the severe situation of the COVID-19 pandemic (Mair-Jenkins et al., 2015).

#### Monoclonal Antibody Therapy

The essence of plasma therapy is antibody therapy. The convalescent plasma of COVID-19 contains various antibodies that can be used to treat antibody-insufficient patients. China has pioneered in treating clinically critical patients using plasma therapy; preliminary clinical trials have shown improved symptoms in many patients. However, plasma therapy is still associated with many uncertainties, such as a limited source of therapeutic plasma and impurities in plasma that may cause safety risks. Relatively speaking, antibody drugs with single ingredients and precise doses are safer and more effective compared with plasma. Among these, monoclonal antibody drugs are the most commonly used. The mechanisms of monoclonal antibody therapy are mainly going to the following four pathways to block the virus from entering the cell: 1) the neutralizing antibody that binds to the S protein on the surface of the virus particle and blocks the binding between the S protein and ACE2; 2) neutralizing antibody that directly binds to ACE2 protein to block the combination of the virus and its receptor; 3) ACE2 analog that competitively binds to S protein on the surface of virus particles with ACE2; 4) antibody against cytokine storm. Apart from combining with the virus, the monoclonal antibody can prevent cytokine storm, which is one of the mortalities for a host of severe COVID-19 patients. Inhibiting interleukin-6 (IL-6), whose secretion leads to an overactive immune response, improves the treatment of severe COVID-19 patients by avoiding cytokine release syndrome (CRS) (Moore and June, 2020). Genentech started to recruit 330 severe COVID-19 patients from April in phase III clinical trial of Tocilizumab (NCT04320615), which is an inhibitor of IL-6. Regeneron Inc. also announced to enroll 400 patients in a clinical II/III trial with Sanofi, treating severe COVID-19 patients with another IL-6 inhibitor, Kevzara (NCT04359901).

### CR3022

**FIGURE 1:**
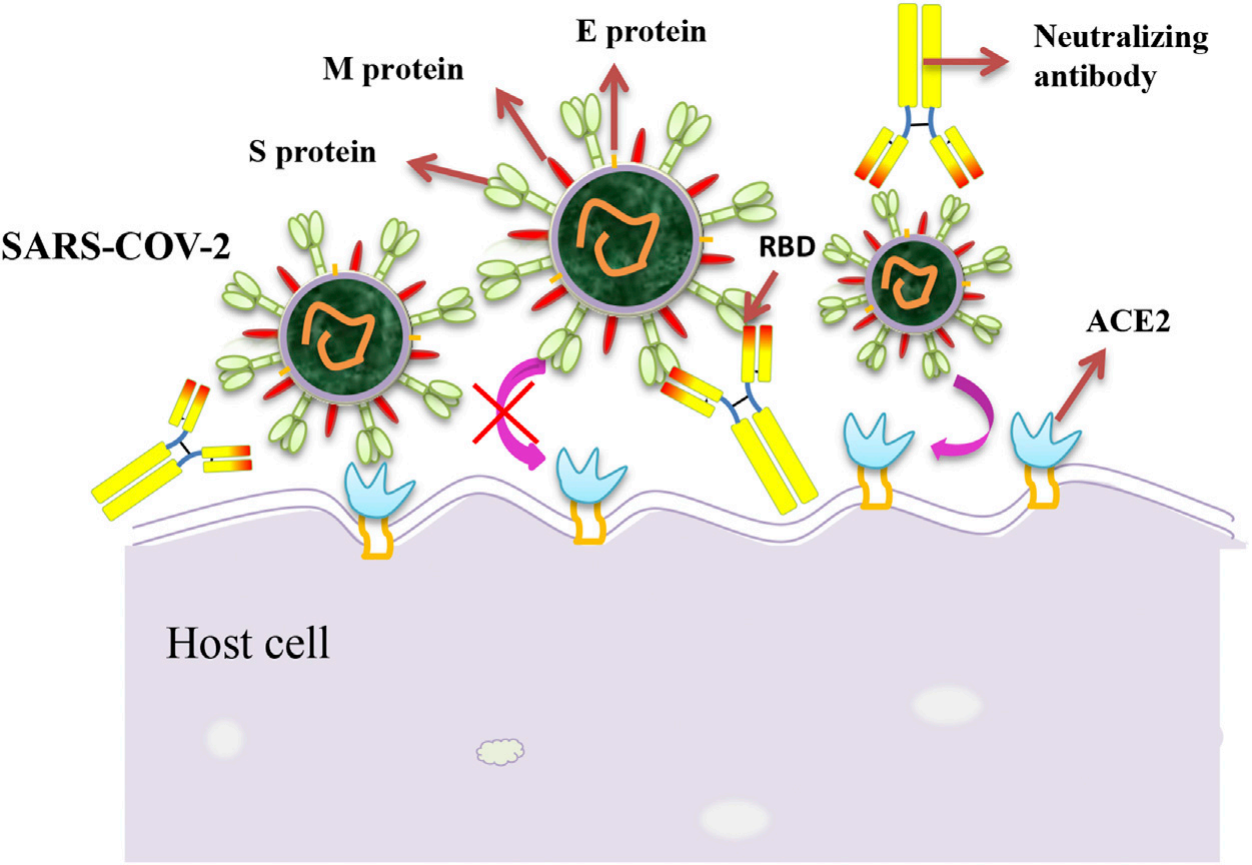
Schematic diagram of the neutralization mechanism of the SARS-CoV-2. The interaction between spike proteins and cell receptors is necessary for membrane fusion and target cell entry. Monoclonal antibodies against the SARS-CoV-2 spike protein may inhibit the binding of the virus to its cellular receptor, thereby preventing the virus from entering the cell.

A previous study found that the receptor-binding domain (RBD) of the SARS-CoV-2 and SARS-CoV had a high similarity (73%) (Lu et al., 2020). Both of them could infect host cells by binding to the receptor angiotensin-converting enzyme II (ACE2). Another study on SARS-CoV revealed that the S protein on the surface of the viral envelope bound to the receptor ACE2 on host cells, thus mediating the adsorption and invasion of viruses (Gallagher and Buchmeier, 2001; Shanmugaraj et al., 2020). Monoclonal antibodies against SARS-CoV may also be involved in SARS-CoV-2 prevention, considering the similarity between the RBD structures of the SARS-CoV-2 and SARS-CoV (Figure 1). Ying Tianlei et al. expressed and purified several specific SARS-CoV antibodies with strong neutralizing activity, including m396 (Zhu et al., 2007), CR3014 (ter Meulen et al., 2004), CR3022 (ter Meulen et al., 2006), and specific human monoclonal antibody m336 for coronavirus of Middle East Respiratory Syndrome (MERS) (Ying et al., 2015). The RBD of SARS-CoV was determined by enzyme-linked immunosorbent assay (ELISA), showing that most SARS-CoV-specific antibodies did not bind to the SARS-CoV-2 RBD obviously except for CR3022, and its binding epitope on the SARS-CoV-2 RBD did not overlap with that of ACE2. This finding indicated that CR3022 had great potential for the prevention and treatment of SARS-CoV-2 infection. The study, which was published online on the preprint platform bioRxiv under the title "Potent binding of 2019 novel coronavirus spike protein by a SARS coronavirus-specific human monoclonal antibody," first reported that the SARS-CoV-specific human monoclonal antibody CR3022 could effectively bind to the SARS-CoV-2 RBD (Tian et al., 2020).

### CD147

On March 14, 2020, a research paper titled “SARS-CoV-2 invades host cells via a novel route: CD147-spike protein” published on the preprint platform bioRxiv by a research team from the Fourth Military Medical University revealed a novel way for SARS-CoV-2 invasion: CD147-S protein (SP) (Wang K et al., 2020). CD147, also known as Basigin or EMMPRIN, is a transmembrane glycoprotein in the immunoglobulin superfamily (Cui et al., 2018). Previous studies showed that CD147 promoted the invasion of SARS-CoV into host cells while CD147 antagonistic peptide-9 inhibited SARS-CoV (Chen et al., 2017). Therefore, the research team investigated the possible role of CD147 in the invasion by novel coronaviruses based on the similarities between SARS-CoV and SARS-CoV-2, and the results confirmed their hypothesis. *In vitro* antiviral tests proved that mepolizumab, a humanized antibody against CD147, could significantly inhibit viral invasion (EC50 was 24.86 μg/ml; IC50 was 15.16 μg/ml) via immunoprecipitation and ELISA. Furthermore, the team used immunoelectron microscopy to observe the localization of CD147 and S proteins in SARS-CoV-2-infected Vero E6 cells (Wang K et al., 2020). All these findings suggested that CD147 might be a key target for specific anti-SARS-CoV-2 drugs.

A clinical trial was conducted by the team subsequently (NCT04275245) to investigate the efficacy and safety of mepolizumab against COVID-19. The enrolled volunteers were divided into two groups. The mepolizumab group was comprised of 17 patients with COVID-19 (4 normal, 6 severe, and 7 critical), while the control group was comprised of 11 hospitalized patients (4 normal, 4 severe, and 3 critical). The baseline characteristics of the two groups were basically balanced. The results were published later on March 24 (Bian et al., 2020). The overall improvement rate, defined as the proportion of patients discharged or with improved severity of illness, significantly improved in the mepolizumab group during the 7-, 14-, 21-, and 28-day follow-ups. The mepolizumab group also showed superiority in discharge and case severity of severe and critical patients compared with the control group. The negative rate and time of viral nucleic acid detection indicated virus clearance, which was significantly shorter (3 days) in the mepolizumab group compared with the control group (13 days). All these data suggested that mepolizumab treatment had a clear benefit in clearing the SARS-CoV-2. Also, the proportion of patients in the mepolizumab group whose lymphocyte counts and C-reactive protein concentrations returned to normal also increased significantly and rapidly. In terms of safety, patients treated with mepolizumab showed no adverse effects. CD147 is also a receptor for the ligand Cyclophilin A (CyPA), and their interaction is critical for inducing inflammation and chemotaxis (Dawar et al., 2017). Accordingly, it was speculated that mepolizumab could also block the interaction between CyPA and CD147, thereby inhibiting the inflammatory response caused by SARS-CoV-2 infection. The anti-COVID-19 ability of mepolizumab cannot be determined at present due to the limited sample size and the nonrandomized nature of the trial. Therefore, large-scale clinical trials are urgently needed to verify the efficacy of mepolizumab.

### 47D11

Both the SARS virus and SARS-COV-2 bind to their receptor ACE2 protein in the S1B domain, which is also the common target of antiviral drug investigations. Wang et al. tried 51 kinds of neutralizing antibodies against SARS hybridoma and examined the 47D11 antibody for its effectiveness against both viruses to determine whether an effective antibody existed against novel coronavirus. For further characterization, they recombined 47D11 into the human IgG1 homologous antibody. The immunofluorescence method detected the direct effect of 47D11 on the SARS virus and SARS-COV-2, while the MERS virus was used for negative comparisons. Neutralizing antibodies can alter the course of infection in the host, thereby enhancing virus clearance or protecting an uninfected host exposed to the virus. Thus, 47D11 has the potential to prevent and treat COVID-19 and possibly also prevent other diseases in the future (Wang C et al., 2020).

### B38 and H4

Some neutralizing antibodies are potential virus-targeting monoclonal antibodies to avoid immune escape. On June 12, B38 and H4 isolated in a convalescent patient with COVID-19 were reported to block the combination between RBD and ACE2, indicating that they might be involved in virus prevention (Wu et al., 2020). Besides, the binding sites of B38 and ACE2 overlapped on RBD while H4 did not, indicating that this pair of antibodies could be used together clinically.

### Narsoplimab

High concentrations of the SARS-CoV-2 prompt the local inflammatory environment and activate the lectin pathway of complement. Hence, patients tend to have endothelial injury and activation during the exudative phase of viral infection, thus leading to a life-threatening ARDS. Narsoplimab, a high-affinity fully human immunoglobulin gamma 4 (IgG4), has been reported to block the lectin pathway by binding to mannan-binding lectin-associated serine protease-2 (MASP-2), which binds to COVID-19 N protein in disease progression. The inhibition of MASP-2, which is located upstream in the lectin pathway, does not cause any difference in an adaptive immune response as the lytic arm of the classical pathway still exists. The results revealed by Alessandro Rambaldi et al. provided a novel sight into COVID-19 immunological therapy via complement inhibition (Rambaldi et al., 2020).

### LY-CoV555

LY-CoV555 (also known as LY3819253), used to be an effective anti-spike neutralizing monoclonal antibody for SARS-CoV-2, was isolated from a blood sample of an early novel coronavirus patient in the United States. The antibody against novel coronavirus spike protein can prevent the attachment and entrance of the virus and is expected to prevent its infection, which also provides another treatment approach for COVID-19. Eli Lilly has completed a phase I study of hospitalized COVID-19 patients (NCT04411628) and a long-term follow-up is ongoing. In the interim analysis of phase II clinical trial (NCT04427501), the 2800 mg dose of LY-CoV555 showed efficacy in viral clearance by day 11 (Chen P et al., 2020). Phase III started in early August to evaluate the safety and efficacy of the developed neutralizing antibody (Lilly, 2020). After the 5-day treatment among 326 participants, the trial was suspended as the difference, which existed between the antibody-treated group and the placebo-treated group, exceeded a predetermined threshold for security. On November 10, the FDA announced a EUA for LY-CoV555 to treat patients aged 12 years and older weighing at least 40 kg who are at high risk of progressing to severe COVID-19 and it was administered as a one-time treatment with intravenous fluids, which provided a new way to prevent novel coronavirus from causing severe symptoms and even death in patients (Chen P et al., 2020).

#### Intravenous Human Immunoglobulin Combined With Low-Molecular-Weight Heparin Anticoagulation Therapy

The treatment mechanism of COVID-19, as a new infectious disease, is still unclear. Cao et al. proposed a reasonable hypothesis on the potential pathogenesis of COVID-19 based on theoretical research and clinical observation and divided the disease progression into three stages: viremia stage, acute stage (pneumonia stage), and convalescent stage (Lin et al., 2020). Viruses enter the lungs and peripheral blood through the mucosa and respiratory tract. If the patient has a good immune function and no other basic diseases, the body can kill the virus quickly and enter the recovery period. If the patient is immunocompromised or damaged and has basic diseases such as diabetes and hypertension, the human immune system cannot eliminate the virus and cause aggravation of the disease. The clinical tests revealed that the number of peripheral blood lymphocytes significantly reduced in severe patients, while the levels of inflammatory cytokines and D-dimer significantly increased. Close to 20% of patients with COVID-19 had abnormal coagulation function, and almost all severe and critically ill patients had coagulation disorders (Chen N et al., 2020; Huang et al., 2020; Wang D et al., 2020). Based on the aforementioned hypotheses and clinical observations on the underlying pathogenesis, the research team suggested that early intravenous human immunoglobulin (IVIG) combined with low-molecular-weight heparin anticoagulation therapy effectively improved the immune function of patients and inhibited the occurrence of the cytokine storm. That is, patients with COVID-19 were immediately treated with adequate doses of IVIG [0.3–0.5 g/(kg ⋅ d) for 5 days] and low-molecular-weight heparin anticoagulation when the number of peripheral blood lymphocytes significantly reduced, the levels of inflammatory cytokines (such as interleukin 6) significantly increased, and coagulation D-dimer levels were four times higher than the upper limit of normal. Also, the laboratory indicators were closely monitored for the side effects after anticoagulation treatment (Lin et al., 2020).

IVIG is a blood product extracted from the plasma of healthy people through a series of complex biotechnologies and administered via intravenous injection. Its main components are broad-spectrum antiviral and bacterial IgG antibodies. After intravenous infusion, the IgG antibodies in the blood of the recipients can be rapidly increased to effectively identify and neutralize pathogens, prevent excessive immunity, maintain immune homeostasis, and cause other effects. A randomized controlled clinical trial on the use of IVIG in treating severe SARS-CoV-2 infection has been initiated (NCT04261426). Despite the excellent efficacy of IVIG in treating influenza (Liu et al., 2016) and SARS (Ho et al., 2004), a large amount of clinical trial data from patients with COVID-19 remain the key to supporting this therapy.

#### Mesenchymal Stem Cell Therapy

Mesenchymal stem cells (MSCs) are characterized by strong anti-inflammatory and immune-regulatory activities, improving the microenvironment of the body and promoting the endogenous repair of the host. The safety and efficacy of MSC therapy have been demonstrated in many clinical trials, especially in immune-mediated inflammatory diseases, such as graft-versus-host disease (Hashmi et al., 2016) and systemic lupus erythematosus (Zhou et al., 2020). Given the strong immune-regulatory capacity of MSCs, the team recruited seven patients with COVID-19 for MSC transplantation to explore the potential of MSCs to treat patients with COVID-19 by observing the changes in immune and inflammatory system functions and the occurrence of side effects within 14 days after transplantation (Leng et al., 2020). MSC transplantation therapy could rapidly and significantly improve the prognosis of severe and critical patients and effectively avoid the cytokine storm, without obvious side effects. This study provided new ideas and hope for treating severe and critical patients with COVID-19, besides hormone therapy and anti-inflammatory therapy.

## Conclusion

In summary, this study was elaborated on the potential immune-related therapies from specific prevention to the prognosis of severe patients (Table 1). These therapies have initially achieved promising results. However, scientific, rigorous preclinical studies and clinical trials are needed to ensure the efficacy and safety of these therapies before they are widely used in treating COVID-19. Research on COVID-19 is still underway globally, and it is believed that the SARS-CoV-2 will be eradicated shortly.

**TABLE 1:** General overview of potential immune-related therapies.

Related therapies			Outline	Research status
Preventive vaccine	Viral vector vaccine	Ad5-nCoV (Adenovirus Recombinant New Corona Vaccine)	Modified human adenovirus type 5 (Ad5) serves as a vector to carry S protein Chen Y et al. (2020), which mediates the invasion of target cells by the new coronavirus and enables the human body to produce immune memory of S protein	Phase I and phase II trials overall progress smoothly; the phase III clinical trial is being conducted
		AZD1222 (ChAdOx1 nCoV-19)	Transformed from attenuated adenovirus, this replicator-defective chimpanzee virus vector carries a transgene that encodes the S protein of novel coronavirus. Patients after vaccination produce antibodies against the virus	Phase I and phase II trials are completed; the phase III clinical trial is being conducted
	mRNA vaccine	BNT162	This is a full-length spike protein mRNA used to induce T cells for better recognition of spikes’ epitopes	Positive results of phase I and part of phase II clinical trials of BNT162b1 are published; a global phase II/III clinical trial has been finished with an efficacy up to 95%
		mRNA-1273	Using lipid nanoparticles (LNPs) as carriers, prefusion of the spike protein stabilizing the SARS-CoV-2 was delivered into the human body to promote human immune response Anderson et al. (2020)	A phase I clinical trial (NCT04283461) was officially launched on March 16 to assess the safety and immunogenicity of mRNA-1273 Laura and Johnson (2020). The phase II and (NCT04405076) III clinical trials (NCT04470427) are being conducted
	DNA vaccine	INO-4800	Insertion of specific protein antigen genes into eukaryotic expression vectors, direct injection, or liposome encapsulation into the body induces specific humoral and cellular immune responses, thereby activating the body’s immune system	INOVIO is the first new coronavirus DNA vaccine to enter clinical trials. Once the safety and immunogenicity data related to the phase I clinical study are available (NCT04336410), INOVIO will promote the phase I/II clinical effectiveness study as soon as possible (NCT04447781)
		GX-19		It entered clinical trials on June 19; phase IIa clinical trials among 150 participants (including placebos) will be started to assess the safety and efficacy of GX-19
	Inactivated vaccine	PiCoVacc	A majority of antigens and epitopes of the virus are aimed at conserved epitopes, thus reducing virus escape and broadening a possible neutralization capability for the virus circulating globally Gao et al. (2020)	It is the first published inactivated COVID-19 vaccine preclinically; phase I and phase II clinical trials are being conducted
	Recombinant protein vaccine	NVX-CoV2373	Coupled with the unique saponin-based Matrix-M adjuvant, NVX-CoV2373 stimulates antigen presentation in local lymph nodes, enhancing the immune response and high levels of neutralizing antibody. In preclinical trials, NVX-CoV2373 blocks the combination between spike proteins and virus-targeted receptors	Phase I trials are completed; phase I/II clinical trial is being conducted
Therapeutic options	Corticosteroids		Corticosteroid, a potent anti-inflammatory, antifibrotic, and vasoconstrictive drug, has been previously applied in many other pulmonary infections to alleviate the condition of patients. It is also potential to be applied in approaches to COVID-19	A large open-label randomized trial recruited 6425 hospitalized patients with COVID-19 carried out in the United Kingdom, and mortality at 28 days showed the superiority in the dexamethasone group. The World Health Organization (WHO) also performed a meta-analysis (PROSPERO CRD42020197242) and then issued living guidance for the use of corticosteroids in severe COVID-19 cases on September 2 WHO (2020b)
	Convalescent plasma therapy		The plasma, serum, or immunoglobulin of convalescent patients with COVID-19 was extracted, and the passive immunity was obtained through the input of the external antibody, to kill the virus or pathogen *in vivo* and achieve the purpose of treating patients	The results showed that the antibodies in the plasma of the convalescent patients may help eliminate the virus and improve the symptoms of patients Shen et al. (2020). However, the sample size of this test is small, no control group is used, and the impact of the timing of plasma infusion is not clear. Therefore, these observations need to be evaluated in clinical trials
	Monoclonal antibody therapy	CR3022	Given the similarity between the RBD structures of the SARS-CoV-2 and SARS-CoV Lu et al. (2020), the research team purified several specific antibodies with strong neutralizing activity and targeting the SARS-CoV’s RBD to study their binding to RBD of SARS-CoV ter Meulen et al. (2004), ter Meulen et al. (2006), Zhu et al. (2007), Ying et al. (2015)	CR3022 has a strong binding ability, indicating that it has great potential to be developed for the prevention and treatment of 2019-nCoV infection Tian et al. (2020)
		CD147	SP can bind to the receptor CD147 on host cells and thus mediate virus invasion Chen et al. (2017), Wang K et al. (2020). CD147 may be a key target for the development of specific anti-SARS-CoV-2 drugs	The results of the clinical trial (NCT 04275245) showed that the anti-CD147 humanized antibody mepolizumab could significantly inhibit the invasion of the virus into host cells Bian et al. (2020). Because the sample size of this study is small, and it is not a randomized controlled trial, the anti-COVID-19 ability of mepolizumab cannot be confirmed at present. A larger clinical trial is needed to verify the efficacy of mepolizumab further
		47D11	The immunofluorescence method detected the direct effect of 47D11 on the SARS virus and SARS-COV-2. Neutralizing antibodies can alter the course of infection in the host, thereby enhancing virus clearance or protecting an uninfected host exposed to the virus	Wang et al. tried 51 kinds of neutralizing antibodies against SARS hybridoma and picked out 47D11 antibody for its effectiveness against both viruses to determine whether an effective antibody existed against novel coronavirus, indicating its potential to prevent and treat COVID-19 and possibly also prevent other diseases in humans in the future Wang C et al. (2020)
		B38 and H4	Some neutralizing antibodies are potential virus-targeting monoclonal antibodies to avoid immune escape. The binding sites of B38 and ACE2 in receptor-binding domain (RBD) on the virus surface overlap, while H4 binds to other sites, indicating that this pair of antibodies can be used together clinically	On June 12, B38 and H4 isolated in a convalescent patient with coronavirus disease 2019 (COVID-19) were reported to block the combination between receptor-binding domain (RBD) and angiotensin-converting enzyme 2 (ACE2), meaning that they might be involved in virus prevention Wu et al. (2020)
		LY-CoV555	LY-CoV555, a lead anti-spike neutralizing monoclonal antibody affinitive to SARS-CoV-2, was obtained from a Covid-19 patient. It was developed by Eli Lilly after AbCellera’s discovering	Phase I study of hospitalized COVID-19 patients (NCT04411628) has been completed and a long-term follow-up is ongoing. Phase II clinical trial (NCT04427501) showed that 2800 mg dose of LY-CoV555 tended to accelerate the viral decline by day 11. Phase III started in early August and stopped on October 13 considering its safety concern. On November 10, the FDA announced an EUA for LY-CoV555
Therapeutic options	IVIG combined with low-molecular-weight heparin anticoagulation therapy		Clinical detection found that the number of peripheral blood lymphocytes in severe patients decreased significantly, while the level of inflammatory cytokines and D-dimer increased significantly Huang et al. (2020), Wang D. et al. (2020). The research team suggested that early IVIG combined with low-molecular-weight heparin anticoagulation therapy could effectively improve the immune function of patients and inhibit the occurrence of the cytokine storm Lin et al. (2020)	A randomized controlled clinical trial has been started (NCT 04261426)
	Mesenchymal stem cell therapy		It is expected that the prevention and reversal of the cytokine storm will become the key to rescuing severe patients with COVID-19 because of its strong anti-inflammatory and immune-regulatory effects, an improvement in the microenvironment, and promotion of the endogenous repair of the host Hashmi et al. (2016), Huang et al. (2020), Zhou et al. (2020)	It was found that mesenchymal stem cell therapy improved the prognosis of severe and critical patients rapidly and significantly, avoided cytokine storm effectively, and had no obvious side effects, thus providing new hope for the treatment of severe and critical patients with COVID-19 Leng et al. (2020)

## Author Contributions

YP and GL conceived the study, reviewed and helped to draft the manuscript. CY and RL drafted the manuscript.

## Conflict of Interest

The authors declare that the research was conducted in the absence of any commercial or financial relationships that could be construed as a potential conflict of interest.
